# GO Nanosheets: Promising Nano Carrier for the S29, 1-(2-Chloro-2-(4-chlorophenyl-ethyl)-*N*-(4-fluorobenzyl)-1*H*-pyrazolo[3,4-d] pyrimidin-4-amine, Therapeutic Agent in Neuroblastoma

**DOI:** 10.3390/ijms21176430

**Published:** 2020-09-03

**Authors:** Stefania Mardente, Michele Aventaggiato, Emanuela Mari, Antonio Francioso, Marco Tafani, Luciana Mosca, Alessandra Zicari, Igor Malyshev, Larisa Kuznetsova, Federica Valentini

**Affiliations:** 1 Department of Experimental Medicine, Sapienza University of Rome, viale Regina Elena 324, 00161 Rome, Italy; michele.aventaggiato@uniroma1.it (M.A.); emanuela.mari@uniroma1.it (E.M.); marco.tafani@uniroma1.it (M.T.); alessandra.zicari@uniroma1.it (A.Z.); 2 Department of Biochemical Sciences, Sapienza University of Rome, piazzale Aldo Moro 5, 00161 Rome, Italy; antonio.francioso@uniroma1.it (A.F.); luciana.mosca@uniroma1.it (L.M.); 3 Institute of General Pathology and Pathophysiology, Moscow State University of Medicine and Dentistry, Baltijskaya Street 8, Moscow 125315, Russia; iymalyshev1@gmail.com (I.M.); lorakuznetsova@gmail.com (L.K.); 4 Department of Science and Chemical Technologies, Tor Vergata University of Rome, via della Ricerca Scientifica, 1, 000133 Rome, Italy; federica.valentini@uniroma2.it

**Keywords:** SRC family kinases, neuroblastoma, graphene oxide, ROS, cytotoxicity

## Abstract

Graphene oxide (GO) derivatives are reported as a valid alternative to conventional carriers of therapeutic agents, because they have a large surface area, an excellent electrical and thermal conductivity and a great capacity for selective binding of drugs and therapeutics, due to the functionalization of their surfaces, edges and sides. In this work GO nanosheets, synthesized by electrochemical exfoliation of graphite (patent N 102015000023739, Tor Vergata University), were investigated as possible carriers of an anticancer drug, the S29, an inhibitor of a cytoplasmic tyrosine kinase (c-SRC) on a neuroblastoma cell line (SK N BE 2 cells). Neuroblastoma is a heterogenous tumor whose characteristics range from spontaneous regression to aggressive phenotypes that are due to different mutations that often occur in SRC family kinases. Inhibitors of tyrosine kinases are currently investigated for their anti-tumoral effects on aggressive neuroblastomas, but their uptake in cells and pharmacokinetics needs to be improved. In this work S29 was stably conjugated with highly water-dispersible GO nanoparticles. S29/GO complex formation was induced by 1h sonication and its stability was analyzed by chromatography coupled with spectrophotometry and mass spectrometry. The synthesized composite (GO-S29) was delivered into SK N BE 2 cells and its effects on cell viability, production of reactive oxygen species (ROS) and migration were studied. The results show that the compound GO-S29 exerts anti-tumoral effects on the neuroblastoma cell line, higher than both GO and S29 do alone and that GO has an additive effect on S29.

## 1. Introduction

Drug delivery systems with engineered nanoparticles have attracted great interest among oncologists for improving tumor response to therapies and targeting tumor cells in solid tumors, thus reducing drug side effects on healthy cells. Our previous studies [1] have shown that physicochemical properties, exposure times and dosages of graphene oxide (GO) on cells are of crucial importance for reducing cytotoxicity and improving uptake in most cells. Because of its enormous versatility in binding different biomolecules simultaneously, GO is often a carrier of choice for intracellular delivery of drugs in tumors, such as neuroblastoma [2], that are poorly affected by traditional chemotherapy.

Neuroblastoma is the most common childhood solid cancer of different phenotypes. It arises in the peripheral sympathetic nervous system due to mutations in various genes that control cell differentiation and growth. Mutations in MYCN or tyrosine kinase receptors have been found in aggressive types of neuroblastomas that tend to relapse and to be resistant to traditional chemotherapy. For these high-risk neuroblastoma patients, new therapeutic targets are continuously being looked for.

The Src family kinases (SFKs) comprise a subclass of membrane-associated tyrosine kinases including Src, Yes, Fyn, Lyn, Hck, Blk, Fgr, and Yrk, which are activated in response to cellular signals that promote cell proliferation, survival, motility, and invasiveness [3]. Moreover, Src plays an important role in angiogenesis and metastasis [4]. Deregulated Src activity is implicated in the development and progression of several human cancers, including breast, brain, and leukemia [5,6,7]. Given that Src has been known to be an important molecular target in cancer, highly specific pharmaceutical compounds are currently available, and a number of Src inhibitors are being investigated in different tumors [8,9]. Recently, new pyrazolo-3,4 pyrimidine deriving Src kinase inhibitors, binding the ATP pocket of the Src kinase, have been synthesized and demonstrated to have antiproliferative and proapoptotic properties in a broad panel of carcinomas [10]. The S29 1-(2-chloro-2-(4-chlorophenyl) ethyl)-*N*-(4-fluorobenzyl)-1*H*-pyrazolo [3,4-d] pyrimidin-4-amine is able to reduce tumor mass in neuroblastoma but it has an unfavorable pharmacokinetic profile [11,12]. Nanostructured innovative materials seem to be able to improve the pharmacokinetic and pharmacodynamic properties of therapeutics, especially in terms of biodistribution, stability and bioavailability. The aim of the present study is to search for a suitable carrier that could improve the bioavailability of a small interfering molecule, known to be active on neuroblastoma cell growth and migration. To this end, we are reporting on the effect of treatment of neuroblastoma cells with GO and S29, both alone and in combination with each other.

## 2. Materials and Methods

### 2.1. Synthesis and Conjugation of GO Nanosheets with S29

GO was synthesized by electrochemical exfoliation of Highly Oriented Pyrolytic Graphite (HOPG) working electrodes (Good Fellow, USA) according to Patent (N°102015000023739).

One aliquot of electrophoresis buffer containing exfoliated GO was diluted 1:10,000 with phosphate buffered saline (PBS) to obtain a 1mg/mL GO concentration. In order to minimize cytotoxicity induced by GO, we used the minimal concentration that allowed the binding of 10 μM S29. A time and dose response curve performed with different cell lines [13,14] established that 2 μg/mL GO did not induce decrease in proliferation rate or necrosis in SK N BE 2 cells. GO (2 μg/mL) and S29 (10 μM) were dispersed in PBS. S29/GO complex formation was induced by 1h sonication. The resulting dispersion was added to cell monolayers. S29 was kindly donated by prof. Maurizio Botta, University of Siena.

### 2.2. Chromatographic Analysis and Binding of GO with S29

To study the interaction between S29 and GO, different samples were prepared and analyzed with liquid chromatography coupled with UV-vis spectroscopy and mass spectrometry. S29 was dissolved in PBS at 10 μM concentration with or without 2 μg/mL of GO. Prior to the analysis each sample was centrifuged at 14,000× *g* for 30 min at room temperature and the supernatant was analyzed by UPLC to verify the presence of S29 in solution before and after interaction with GO.

UPLC-DAD-MS was performed on a Waters Acquity H-Class UPLC system (Waters, Milford, MA, USA), including a quaternary solvent manager (QSM), a sample manager with a flow through needle system (FTN), a photodiode array detector (PDA) and a single-quadruple mass detector with electrospray ionization source (ACQUITY QDa). Chromatography was performed on a Phenomenex Kinetex C18 column (100 mm × 2.1 mm i.d., 2.6 μm particle size). Solvent A was 0.1% aqueous HCOOH and solvent B was 0.1% HCOOH in MeOH. The flow rate was 0.5 mL/min and the column temperature was set at 35 °C. Elution was performed running the first 2 min isocratically with 2% of solvent B, then applying a 10 min linear gradient from 2% to 100% B. The column was re-equilibrated for 5 min with 98% A and 2% B before the next injection. Samples were filtered before the analysis and 10 μL were injected through the needle. The PDA detector was set up in the range of 200 to 600 nm. Mass spectrometric detection was performed in the positive electrospray ionization mode using nitrogen as nebulizer gas. Analyses were performed in the Total Ion Current (TIC) mode in a mass range of 120–600 *m*/*z*. Capillary voltage was 0.8 kV, cone voltage 30 V, ion source temperature 120 °C and probe temperature 600 °C.

### 2.3. Cell Lines and Culture Conditions

The human neuroblastoma cell line SK N BE(2), obtained from DSMZ, Braunschweig, Germany, SK-N-BE(2) (ACC No 632) was maintained in a humidified incubator with 5% CO2, 37 °C, in DMEM, (Invitrogen, Life Technologies, Monza, Italy) supplemented with 10% FBS, 10 IU/mL of penicillin and 10 mg/mL of streptomycin.

### 2.4. GO Uptake in Cells

Cells (10^5^/mL) were seeded into 6 well plates (Corning Inc., Corning, NY, USA), allowed to attach for up to 6 h, cultured in DMEM, 10% FBS, 10 UI penicillin and 10 mg/mL streptomycin, supplemented with different concentrations of GO for 2 h. Controls were made with untreated cells.

After incubation, cells were washed twice with PBS and fluorescence deriving from GO uptake was evaluated by cytometry (Epics XL-MCL Coulter, Brea, CA, USA) at 495–519 nm, according to [15].

### 2.5. Cell Viability Assay

Cells were seeded in 96-well plates at a concentration of 1 × 10^4^ cells/well and after 24 h treated or untreated with GO and S29 at concentrations of 2 μg/mL and 10 μM, respectively. Controls were made with untreated cells grown under the same experimental conditions.

10 μL of WST 1 assay kit cell proliferation (Abcam, Milano, Italy) solution was then added to each well and microplates were incubated for 2 h in standard culture conditions. After incubation, absorbance was read in a multiwell spectophotometer (DAS, Rome, Italy) at 440 nm.

### 2.6. Reactive Oxygen Species (ROS) Detection

ROS formation in SK N BE (2) cells with GO and S29 was assayed by standard methods using DCF-DA dye and flow cytometry [16]. DCF-DA (final concentration of 40 μM) was added to cell cultures on 6-well plates for 15min at 37 °C. After incubation, cells were scraped, washed in PBS, and analyzed by a flow cytometer with an argon laser at 488 nm. Cells were gated using forward angle light scatter (FS) and 90° light scatter parameters (SS). For every histogram, a minimum of 20,000 events were counted. The mean fluorescence intensity was detected and expressed as a percentage of relative ROS level versus control cells.

### 2.7. Cell Cycle with PI

Cell cycle was analyzed by flow cytometer, using the fluorescent nucleic acid dye propidium iodide (PI) (Epics XL-MCL Coulter, CA, USA). Cells were washed twice in phosphate buffered saline (PBS) and fixed in 70% ethanol in PBS for 1 h at 4 °C. Then, cells were then washed twice with PBS, resuspended in 0.5 mL PBS, 50 µL RNase A (5 µg/mL) (Sigma-Aldrich, St. Louis, MI, USA), and stained with 0.5 mL of 100 mg/mL PI (Sigma-Aldrich) in PBS. Cells were incubated for 30 min at room temperature in the dark and analyzed for DNA content. The fluorescence was measured using an EPICS profile cytometer.

### 2.8. Wound Healing Assay

The wound healing assay was performed with Ibidi 2 well inserts mounted on a 24 well plate (Ibidi GmbH, Gräfelfing, Germany).

Cells were allowed to adhere to multiwell plates containing the inserts with defined 500 µm gaps. After 4 h adhesion, the inserts were removed and the monolayers were photographed at different time intervals.

### 2.9. Statistics

All statistical analyses were performed using KaleidaGraph version 4.5.1 (Synergy Software Inc., Reading PA, Eden Prairie, MN, USA). Data are the mean of three independent experiments. All measurements were performed in triplicate, expressed as mean values ± standard deviation and analysed using Student’s *t*-test. *p* < 0.05 was considered to indicate a statistically significant difference.

## 3. Results

### 3.1. Synthesis of GO and Conjugation with S29

**Figure 1:**
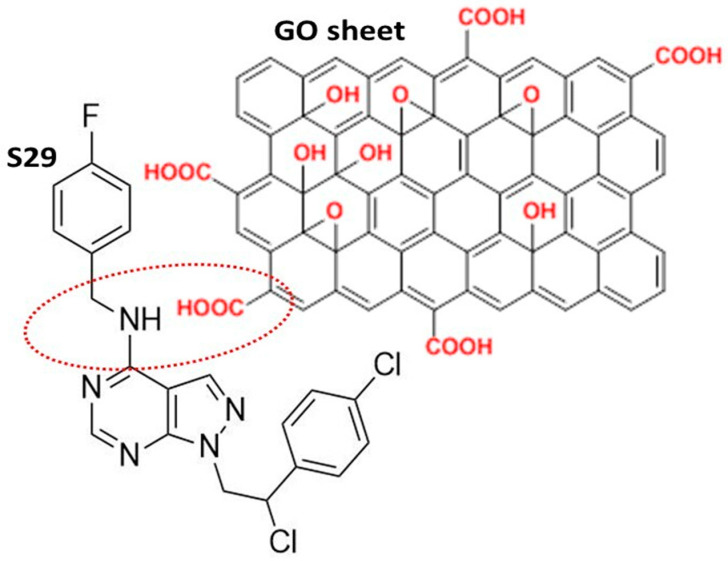
Schematic representation of the most likely interaction between S29 and the graphene oxide (GO) nano sheet.

The GO used in this work was a high-quality double layer of graphene, containing oxygenated functional chemical groups, responsible for the efficient binding of S29 (Figure 1). The chemical and physical properties of GO are summarized in Table 1 [17].

**Figure 2:**
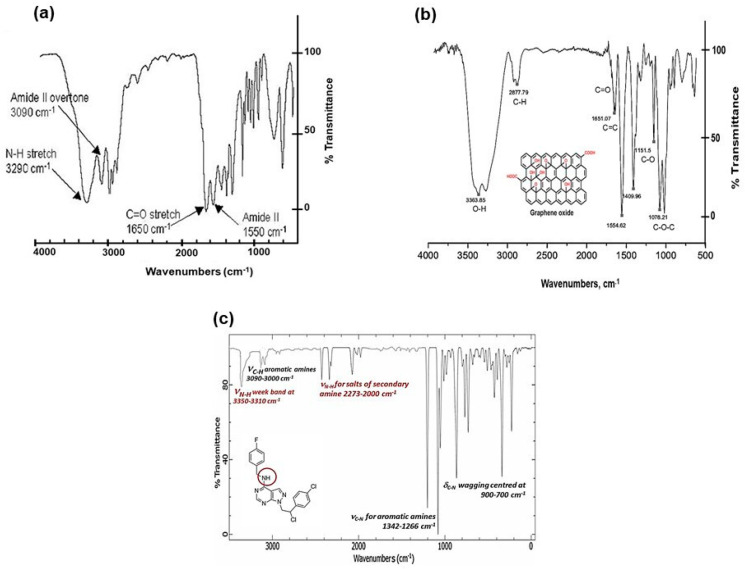
(**a**) FTIR spectral profile of GO/S29 composite materials (GO-S29); (**b**) FTIR spectral profile of GO; and (**c**) FTIR spectral profile of S29 molecule.

The FTIR (Fourier Transform Infra-Red spectroscopy) profile revealed the typical secondary amide fingerprint, with the specific band assignments highlighted in the FTIR spectrum of Figure 2. The amide adsorption wavelengths (cm^−1^) were shifted at lower IR values for aromatic rings in the S29 molecular structure (Figure 2a), that stabilize the molecular system by electronic resonance. Considering the alkaline feature of the secondary amines in S29 (-NH-) and the acidic nature of the carboxyl groups (-C(=O) OH-) in GO, an amide-like bond seems most likely because it occurs at physiological conditions (PBS, pH = 7.4, R.T). Other interactions between Cl, F N=N groups in S29 and alcohols, ethers and/or epoxy groups on GO nano-sheets do not occur spontaneously as a result of the above process and would only be possible with specific chemical reagents (not applied in this work).

Figure 2b, shows a GO sample (used here as a control), functionalized with alcoholic and carboxylic organic groups. Figure 2c, shows a FTIR profile of S29 with secondary and tertiary (i.e., aza groups) aromatic amines.

Other interactions between Cl, F N=N groups in S29 and alcohols, ethers and/or epoxy groups on GO nano-sheets do not occur spontaneously as a result of the above process and would only be possible with specific chemical reagents (not applied in this work). The Z-potential value of final composite (GO-S29- ξ = −37.80/mV) is very similar to that of GO-CTRL (ξ = −38.47/mV). This means that the amide-covalent bond does not significantly alter the electrostatic polarity of the final composite material. The partial separation of electrostatic charges, established between the amidic nitrogen and the carbonyl groups in amide bonding, is responsible for the partial polar features of the typical covalent amide bond.

FTIR results were also confirmed by XPS (X-Ray Photoelectron Spectroscopy) data, where the C1s signal (centred at 287.84 eV) was assigned to the amide bond –N–(C=O). The relevant N1s region was fitted by one peak assigned to –N–(C=O) (400.50 eV). Both C1s and N1s analytical signals unequivocally demonstrated the typical amide bond in the GO-S29 composite material [18,19], (XPS data shown in [1] and summarized in Table 2.

### 3.2. Determination of Binding and Stability of GO-S29 Conjugates

**Figure 3:**
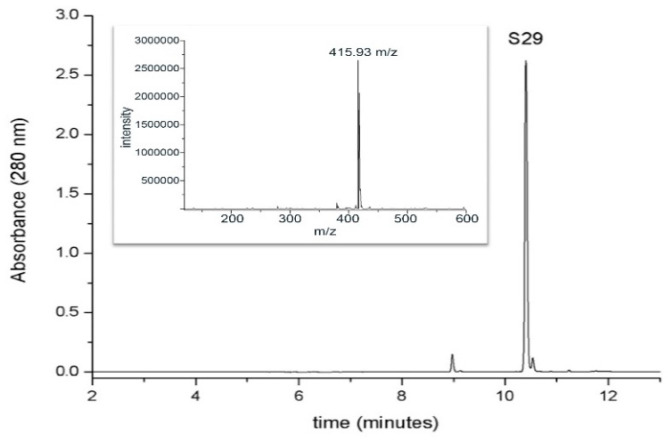
Chromatographic analysis of S29UPLC profile (at 280 nm) of S29 1mM standard solution. The inset shows the mass spectrum of the molecule in positive ion mode (retention time, 10.4 min).

For the detection and the determination of S29, a chromatographic method coupled with spectrophotometric and mass spectrometric detection was used. The chromatographic behavior of S29 (retention time, 10.4 min) and the mass spectrum (ESI positive mode) are shown in Figure 3.

Figure 3 shows the capacity of GO to interact with S29 and form a compound. Each chromatogram represents the amount of free S29 in each sample.

**Figure 4:**
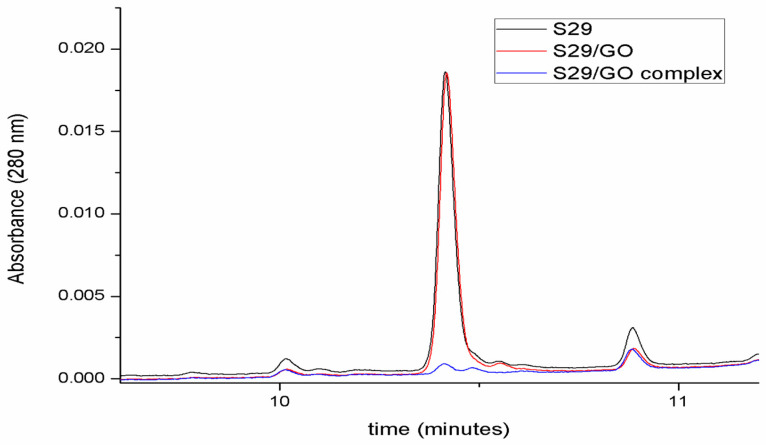
Determination of GO/S29 interaction. Chromatographic analyses of S29 10 μM solution in phosphate buffered saline (PBS) (black), in the presence of 2 μg/mL GO before (red) and after ultrasonication (blue).

The red chromatogram in Figure 4 highlights the fact that GO does not affect the number of free molecules in solution before ultrasonication. After the induction of complex formation by ultrasonication (blue chromatogram in Figure 4), S29 molecules are no longer detectable in solution, underlying an association between GO and S29.

### 3.3. Uptake of GO by SKNBE (2) Cells

We previously demonstrated that GO particles enter the cell walls without damaging the membrane or the nucleus. The uptake of GO in cells was proved by evaluating the fluorescence properties of GO in cytofluorimetry in the band of green fluorescence at 495–519 nm.

**Figure 5:**
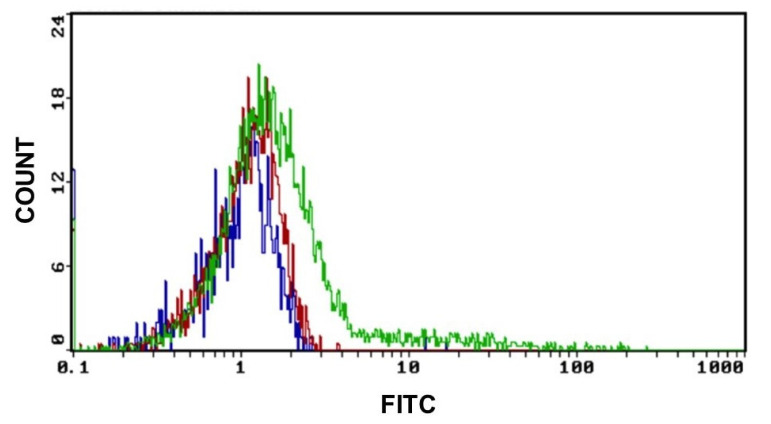
GO uptake indicative fluorescence peaks of GO uptake in cells after 2 h of exposure to 0.2 μg and 2 μg of GO for 2 h. (Blue graph: CTRL, red graph: cells treated with 0.2 μg GO and green graph: cells treated with 2 μg of GO) Excitation and emission spectrum peak wavelengths of approximately 495 nm/519 nm.

As shown in Figure 5, the GO uptake starts after 2 h of exposure at a concentration of 0.2 μg/mL of GO and increases when the working dose of 2 μg/mL is applied. Controls were made with untreated cells.

### 3.4. Additive Effect between GO and S29 in Inducing Cell Death

**Figure 6:**
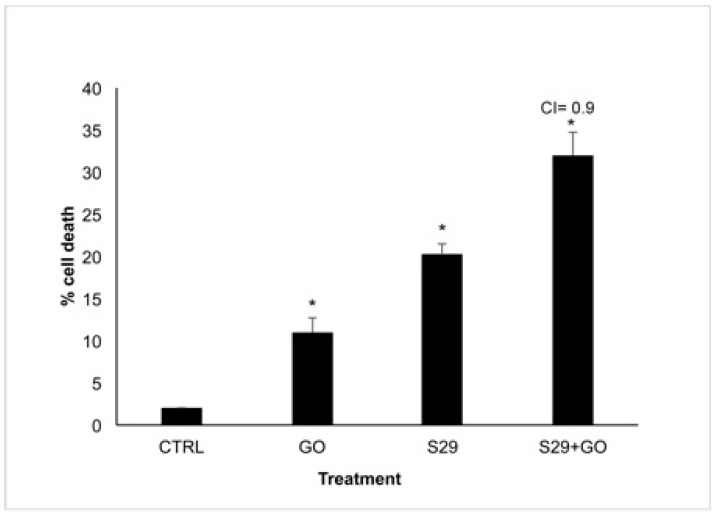
Cell Viability assay SK NBE (2) cells were treated with GO (2 μg/mL), S29 (10 μM) and GO-S29 (2 μg/mL–10 μM) for 24 h. Histograms show % of cell death. Combination index (CI) = 0.9 indicates an additive effect of GO-S29. Results of three independent experiments are shown in histograms. Significant differences versus control are indicated by asterisks (*).

In order to reveal a potential combined effect of GO and S29, we incubated neuroblastoma cell line SKNBE (2) with the minimum effective doses of GO (2 μg/mL) and S29 (10 μM) both individually and in combination with each other for 24 h. The effect of these treatments on reduction in cell viability is shown in Figure 6.

**Figure 7:**
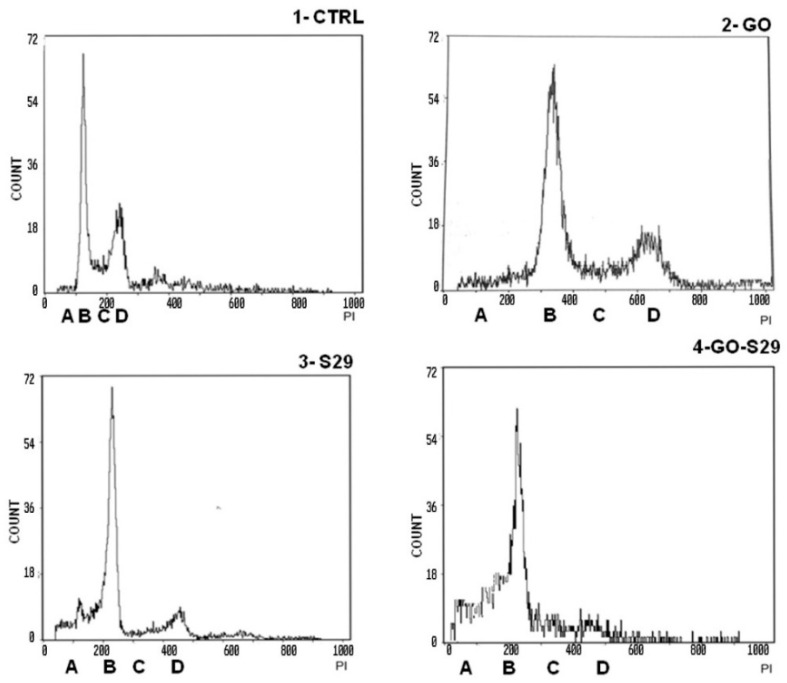
Analysis of cell cycle: Cells were incubated with the described substances for 24 h. The first peak shows a pre-G1 phase A with fragmented nuclei; the second peak B shows the G1 phase, the area C between peak B and peak D shows the S phase; peak D shows G2 M transition. Representative results of three independent experiments are shown in graphical representation (panels A, B, C, D) of the percentage of cells in the different phases of the cell cycle (shown in Table 3) in control and treated cells. The data represent the mean ± standard deviation; * *p* < 0.05 versus control.

Death in cells treated with GO alone is probably due to the high proliferation rate of SK N BE (2) cells. At the above GO concentration, cell death is not induced in normal cells or in any other cell line tested [1]. This result is in line with the slight increase (8%) of cells in pre-G1 phase treated with 2 μg/mL GO for 24 h, compared with untreated controls, shown in Table 3 and Figure 7.

To calculate the combination index (CI), the algorithm described by Fransson et al. [20] was used. We analyzed the effect of the combined GO-S29 compound and compared it with that exerted by the single agents. According to the authors who studied the effects of chemotherapeutic agents used in combination, a CI in the range of 0.8 and 1.2 relates to an additive effect of the two substances.

As shown in Figure 6, addition of the combined compound (GO-S29), yielded an increase in cell death, thus showing an additive effect of the GO-S29 compound. This is also proved by the CI of 0.9 as indicated in Figure 6.

### 3.5. The Combination of GO and S29 Inhibits SKNBE (2) Proliferation

In view of the reported effects on cell viability, we explored the mechanisms responsible for the additive effect of the GO-S29 compound on the DNA synthesis ratio in cells after 24 h treatment with GO and S29 both alone and in combination with each other.

Cell cycle studies of SKNBE (2) cells treated with GO, S29 and GO-S29, summarized in Table 3, show that when combined with GO the proapoptotic and antiproliferative effects (pre-G1/A and G2M/C-D peaks in Figure 6, respectively) of S29 significantly increase (*p* < 0.001) (Figure 7).

There were significant differences between pre-G1 phase and S-G2 phases, between controls, and between cells treated with GO-S29 and with S29 alone. The increase in fragmented nuclei in cells treated with S29 and S29-GO, (see A peaks in panels three and four) is probably due to apoptosis. As well as an increase in the pre-G1 phase of the cell cycle, GO-S29 induces a dramatic decrease in the G2-M phases, indicating a very low proliferation.

### 3.6. The Combination of GO and S29 Induces ROS Production in SKNBE (2) Cells

We evaluated the oxidative pathway in cells treated with GO and S29 as a potential mechanism involved in cell cycle arrest and cell death induction since it is known that ROS could exert cytotoxic activity and induce DNA damage and apoptosis [21]

**Figure 8:**
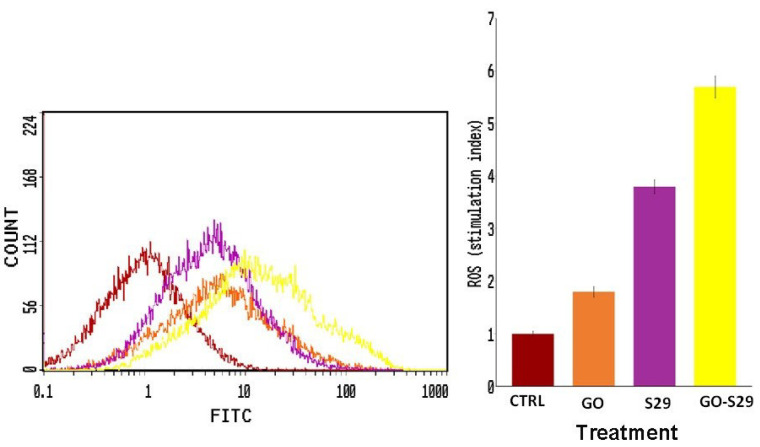
Reactive oxygen species (ROS) production: ROS were detected by flow cytometry; the mean fluorescence intensity was detected and expressed as a stimulation index obtained by the ratio of ROS levels released by cells after 2 h of treatment and ROS detection in control cells. Data are the mean + SD of 3 different experiments. Indicative fluorescence peaks of differently treated cells (shown on the left of the figure).

ROS production in cells treated with GO and S29 was assayed by flow cytometry. As shown in Figure 8, S29 delivered by GO induces a higher production of ROS compared to GO or S29 alone. The percentage of cells producing ROS is shown in (Figure 8).

### 3.7. The Combination of GO and S29 Inhibits SKNBE (2) Migration

**Figure 9:**
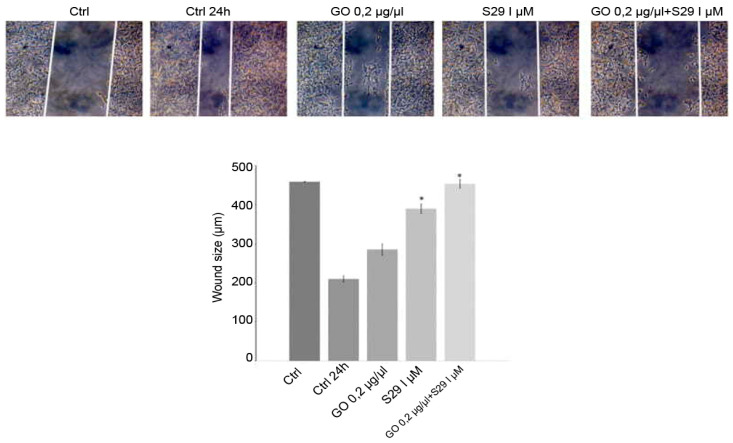
Wound healing assay: Cells were cultured with different treatments for 24 h in wound healing assay chambers (Ibidi, UK). Cells were cultured with different treatments for 24 h in wound healing assay chambers (Ibidi, UK). Photographs were taken at 10x magnification with a Motic AE31 microscope (Motic Europe, Spain) after adherence (CTRL) and at 24 h. Photograms were taken with a Nikon Digital Camera DS-L1 (Nikon Corporation US) and analyzed with the custom software. Representative results of three independent experiments are shown in histograms. Significant differences versus control are indicated by asterisks (*). (S29 versus CTRL 24 h *p* = 0.002; GO-S29 versus CTRL 24h *p* = 0.001; S29 versus S29-GO *p* = 0.04).

The wound closure assay showed that GO-S29 significantly conjugates suppressed migration of SK N BE (2) cells. Figure 9 shows that in 24 h controls and in cells cultured with GO or S29 alone, the size of the wound gets smaller, suggesting that the cells invade the scratch in the culture plate, whereas in cells treated with GO-S29, the size of the scratch becomes larger, almost as large as in time 0 controls. The wound size in cells treated with S29 alone and GO-S29 is significantly higher compared to 24 h controls.

## 4. Discussion

Since only a few treatment options are available for neuroblastoma with an aggressive phenotype, novel therapeutic strategies are needed.

Tyrosine kinases are receptors that 25 years ago were found to function as oncogenes. Only recently were their inhibitors used in anticancer therapy. They are used in leukemias, but their use in solid tumors needs to be improved [22].

Tyrosine kinases are enzymes that catalyze the phosphorylation of tyrosine residues. Receptor TKs have an extracellular, a transmembrane and an intracellular domain. When the extracellular domain is bound by a ligand, like a growth factor, the intracellular tyrosine residues autophosphorilate and activate the transduction pathway leading to the translation of proliferative and anti-apoptotic genes [23]. Small molecule inhibitors may either block the ATP binding site in the intracellular domain in order to prevent phosphorylation or, as in the case of monoclonal antibodies, block the extracellular binding site.

TK inhibitors are used in combination with radiotherapy in leukemias. S29 (imatinib) [24,25] is a small molecule TK inhibitor that has been shown to have a positive effect in reducing neuroblastoma in mice models. The problem with solid tumors is the low bioavailability of these drugs and their high cost [26]. Different nanoparticles have been designed for direct targeting tumor cells but the difficulties of interacting with the tumor microenvironment must be taken into account [27], as well as the cytotoxic effects that nanomaterials might exert on normal healthy cells, or even the degradability of the formed structures which depend on the purity and size of the material [28].

Size, shape, and charges of the nanoparticle must be taken into account in the design of drug delivery nanocarriers. Although there is not a universal ‘adequate’ size for optimal nano particle internalization, this may influence both the endocytic pathway followed for internalization into cells and cytotoxicity [29].

Many authors have shown that small sized nanoparticles and the medium used for their dispersion, can implement diffusion and adhesive interaction with the cell membrane lipid bilayer [30,31,32], minimizing the risk of damaging cell membranes. In the case of carbon derived nanomaterials, it has been demonstrated [29] that interaction of nanoparticles with cells leads to modifications of membrane fluidity that facilitate their internalization without affecting membrane integrity or even causing the formation of pores. The qualities of GO as drug carrier have been stressed by many authors [33] for its capacity to be functionalized with different molecules that are directly pharmacologically active or enhance selective binding to receptors or cell surfaces [34,35]. Functionalization of graphene could occur through both covalent and noncovalent approaches to modify its physical and chemical properties [36]. In the case we are reporting here of GO and S29, an amide bond is formed that was stable under physiological conditions. Other authors [36] demonstrated that the covalent binding of doxorubicin with carbon derived nanomaterials, allows less efflux of drug from cells, compared with ionic bonds, suggesting that the longer time of exposure to drugs may result in a more pronounced anti-tumoral effect.

One strategy for synergizing with anti-cancer agents is to induce ROS mediated cytotoxicity, [37] as they may induce apoptosis, we found here that ROS production in cells treated with GO alone was very low but it increased when combined with S29, thus contributing to the enhancement of the cytotoxic effect of GO-S29.

Tumor cell migration is an important part of the metastatic process that is the result of a more aggressive phenotype acquired during cancerogenesis [38,39]. We have shown that the composite GO-S29 reduces neuroblastoma cell migration.

In conclusion, a chemotherapeutic system that is able to reduce growth and prevent invasion of tumor cells is a promising agent for the treatment of neuroblastoma. GO used at the lowest concentration needed to carry the minimum effective dose of S29, might be an efficient and effective nanocarrier for a small interfering anti-TK molecule into tumor sites.

## Figures and Tables

**Table 1:** Physico–chemical Properties of synthesized GO.

Elemental Analysis (% *w*/*w*) ^a^	Si n.d. S n.d. Ca n.d. Cr n.d. Fe n. d. Co n. d.
Thickness (nm) ^b^	(1.20 ± 0.30; a bilayer)
Area (nm^2^) ^c^	(0.10–3.00)
weight loss% (TGA) ^d^	(2.80 ± 0.50)
acidic sites (nmol/mg) ^e^	(63.22 ± 2.70)
extent of defect (I_D_/I_G_) ^f^	0.10
Z-potential (ξ/mV)	−38.47

^a^ Evaluated by XRF spectroscopy (mean ± SD). ^b^ Evaluated by AFM analysis (mean ± SD). ^c^ Evaluated by AFM analysis (mean ± SD). ^d^ Evaluated by Thermo-Gravimetric Analysis (mean ± SD). ^e^ Evaluated by the volumetric titration of the acidic sites [17] (mean ± SD). ^f^ Evaluated by Raman spectroscopy. n.d. means non detectable elements, because the values are below the sensitivity parameter of the XRF techniques.

**Table 2:** Spectroscopic characterization of GO and GO/S29 composite materials. |

GO			GO/S29 Composite		
**FTIR**			**FTIR**		
Carboxylic acids, C(=O)OH: *ν_OH_* 3300-2500 cm^−1^_;_ *ν_C(=O)_*1717 cm^−1^; *δ_-in-p-C-O-H_* 1424 cm^−1^; *ν_-C-O_* 1301 cm^−1^; *δ_-out-of-p- -O-H_* 946 cm^−1^			Amide, $\begin{array}{l} -C\left(=O\right)-N-R:\\ \;|\\ \;H \end{array}$ *ν_C_*_(=O)N_ 1550 cm^−1^		
Chetons, C(=O)R: normal *ν_C(=O)_* 1715 cm^−1^; *ν* and *δ*_C-CO-C_ 1213 cm^−1^ Aldheids, C(=O)H: *ν_C_*_-H_ 2715 cm^−1^; *ν_C_*_(=O)_ 1728 cm^−1^; δ_C-H_ 1381 cm^−1^			Amide, $\begin{array}{l} -C(=O)-N-R:\\ \;|\\ \;H \end{array}$ *ν_C_*_=(O)_ 1650 cm^-1^		
Ethers, -C-O-C: asymmetrical *ν_C_*_-O-C_ 1125 cm^−1^			Amide, Overtone, $\begin{array}{l} -C\left(=O\right)-N-R:\\ \;|\\ \;H \end{array}$ *ν_C_*_(=O)N_ 3090 cm^−^^1^		
Alcohols, C-OH: *ν_OH_* 3350 cm^−1^; *ν_C_*_-O_ 1054 cm^−1^			Amide, $\begin{array}{l} -C\left(=O\right)-N-R:\\ \;|\\ \;H \end{array}$ v_NH_ 3290 cm^−1^		
**XPS**			**XPS**		
*Functional groups*	*Peak Binding Energy (eV)*	*At (%)*	*Functional groups*	*Peak Binding Energy (eV)*	*At (%)*
C(=O)O	289.3	5.0	Amide **–N–(C=O)**	C1s 287.84	10.0
C(=O)	287.8	8.0			
C-O	286.5	16.0	Amide **–N–(C=O)**	N1s 400.50	7.0
C-OH	285.2	18.0			
*At (%): Atomic percentage (%)*					

**Table 3:** Analysis of the cell cycle.

SKN BE (2)	Pre-G1 (A)	G0-G1(B) (Dyploid)	G2-M (C-D) (Hyperploidy)
**CTRL**	5 ± 6%	67 ± 8%	28 ± 9%
**S29 10 μM**	16 ± 6%	72 ± 6%	12 ± 5%
**GO 2 μg/mL**	8 ± 4%	62 ± 2%	30 ± 6%
**GO 2 μg/mL + S29 10 μM**	38 ± 7%	60 ± 3%	2 ± 2%

% of cells in the indicated phases of the cell cycle. Cells were incubated with the described composites for 24 h. At the end of incubation cells were stained with propidium iodide (PI) before cytometry at 480 nm. Experiments were performed in triplicate, data are the mean values ± SD.
